# Antioxidant and Antiproliferative Activities of Several Garlic Forms

**DOI:** 10.3390/nu15194099

**Published:** 2023-09-22

**Authors:** Zeinab Farhat, Tyler Scheving, Diana S. Aga, Pamela A. Hershberger, Jo L. Freudenheim, Rachael Hageman Blair, Manoj J. Mammen, Lina Mu

**Affiliations:** 1 Department of Epidemiology and Environmental Health, State University of New York at Buffalo, Buffalo, NY 14260, USA; zfarhat@buffalo.edu (Z.F.); jfreuden@buffalo.edu (J.L.F.); 2 Department of Chemistry, College of Arts and Sciences, State University of New York at Buffalo, Buffalo, NY 14260, USA; tjschevi@buffalo.edu (T.S.); dianaaga@buffalo.edu (D.S.A.); 3 Department of Pharmacology and Therapeutics, Roswell Park Comprehensive Cancer Center, Buffalo, NY 14263, USA; pamela.hershberger@roswellpark.org; 4 Department of Biostatistics, State University of New York at Buffalo, Buffalo, NY 14260, USA; hageman@buffalo.edu; 5 Department of Medicine, State University of New York at Buffalo, Buffalo, NY 14260, USA; mammen@buffalo.edu

**Keywords:** lung cancer, garlic extracts, antioxidant, antiproliferative, chemoprevention

## Abstract

It is hypothesized that garlic, *Allium sativum*, might protect against oxidative stress that causes damage to cells and tissues leading to the development of various health conditions including cancer. However, it is not known whether garlic’s potential anticancer benefits differ by form of garlic consumed. This study aimed to quantify and compare the in vitro antioxidant and antiproliferative activity of several garlic forms in water and alcohol extracts including fresh garlic, fresh garlic set aside, heated garlic, heated garlic set aside, garlic powder, black garlic, two commercially available garlic supplements. Antioxidant activity of different garlic forms were measured using three assays: DPPH (2,2-diphenyl-1-picryl-hydrazyl-hydrate) assay, superoxide assay, and hydroxyl assay. In vitro effects of garlic extracts were investigated against the most common lung cancer subtypes: H520, H1975, and A549 cell lines using the sulforhodamine B (SRB) assay. Among free radical scavenging assays, Garlicin^®^, a commercially available supplement, displayed high antioxidant activity in water and alcohol extracts (DPPH assay: 2.02 mg AAE (mg ascorbic acid equivalent)/g garlic and 3.53 mg AAE/g garlic, respectively; superoxide assay: 6.73 mg AAE/g garlic and 7.13 mg AAE/g garlic, respectively). In the hydroxyl assay, water extract of fresh garlic crushed and set aside for 10 min showed the highest antioxidant activity. Garlicin^®^ alcohol extract and fresh garlic water extracts strongly inhibited the proliferation of H1975, A549 and H520 cells. Other forms of garlic including garlic powder and black garlic exhibited low antioxidant and antiproliferative activity. Our results demonstrate that the preparation and processing methods of garlic may lead to different antioxidant benefits.

## 1. Introduction

*Allium* vegetables, including garlic, are rich sources of several phytochemicals with known health benefits [1]. The beneficial properties of garlic are mainly attributed to their high content of organosulfur compounds, including alliin, allicin, thiosulfates, and water-soluble S-allylcysteine (SAC) and S-allylmercaptocysteine (SAMC) [2]. These compounds have potent antioxidant activity and contribute to reduced oxidation by radical scavenging activity and to enhanced antioxidant enzymes’ activity.

The chemistry of garlic is complex; variation in processing can yield different compounds, with differences in potency. As normally used in Western diets, it is frequently processed and heated. The chemicals present in garlic products largely depend on manufacturing conditions, temperature and duration of drying, and polar and/or nonpolar extraction solvents [3]. These differences may explain inconsistency in results of previous observational and clinical studies of dietary garlic consumption and colorectal, gastric, prostate, breast, and lung cancer [4]. Different garlic forms have been shown to have significant antioxidant effects in experimental and human studies [5,6,7,8]. Since the processing method of garlic results in structurally altered chemical constituents, it is critical to identify which garlic forms contain the most abundant and effective anticancer components. In experimental studies, dietary supplementation of garlic and its derivatives have been shown to result in markedly lower intracellular ROS and upregulation of detoxifying enzymes and enzymes involved in antioxidant activity [9,10,11,12,13]. In randomized trials, there is evidence garlic supplementation may contribute to increased lead to a beneficial increase of antioxidant capacity [8,14,15].

Commonly used garlic forms include raw garlic, cooked garlic, garlic powder, aged garlic, and garlic supplements. Whole raw garlic contains γ-glutamyl-S-allyl-L-cysteines: this compound, when hydrolyzed and oxidized, yields alliin, an organosulfur compound. Upon chewing or crushing whole raw garlic, alliinase, an enzyme found in the cell wall, is activated, and converts alliin into allicin. When set aside within 10 min, allicin quickly decomposes into sulfur compounds, including diallyl sulfide (DAS), diallyl disulfide (DADS), and diallyl trisulfide (DATS) [16]. Cooking or even heating garlic may reduce some of the bioactive compounds present in garlic [17]. The alliinase enzyme can be deactivated by heat. Microwaving garlic can block the ability of garlic to reduce the formation of DNA adducts, which can lead to mutations and give rise to cancer [18].

Lung cancer remains the most common and fatal cancer in the world [19]. Preclinical, epidemiological, and clinical studies have suggested some benefits of garlic intake regarding lung cancer incidence [20,21]. However, results remain inconsistent because of there is differentiation in individual garlic products in these studies.

It is critical to identify which garlic forms contain the most abundant and effective antioxidant and antiproliferative activity. Most in vitro studies have focused on one garlic form or one specific garlic compound, limiting comparisons of the antiproliferative effects of different garlic forms in the same study. We report here on a study comparing antioxidant and antiproliferative activity for several common garlic products (raw/fresh crushed, raw/fresh garlic crushed and set aside for 10 min, garlic powder, cooked/heated garlic, cooked/heated garlic crushed and set aside for 10 min, black garlic, and two garlic supplements) and in different extraction solvents (alcohol and water).

## 2. Materials and Methods

### 2.1. Garlic Sources

Garlic was purchased from a single local grocery market in Buffalo, NY, USA. The garlic forms chosen were crushed fresh garlic, fresh garlic crushed and set aside for 10 min, garlic heated for 60 s on high in a 4000-watt microwave, garlic crushed set aside and then heated 60 s on high in a 4000-watt microwave, garlic powder, aged black garlic, and two garlic commercial supplements (Garlicin^®^ and Kyolic). Garlicin^®^ (Nature’s Way, Green Bay, WI, USA) was selected because it is the only supplement that has been shown in bioavailability studies to release allicin at a level equivalent to that of crushed raw garlic [22]. Kyolic^®^ (Wakanuga Pharmaceutical, Mission Viejo, CA, USA) was selected because it is a popular brand of garlic supplement that contains a different form of garlic (aged) shown to have lipid-lowering effects in clinical studies [23,24]. Fresh garlic (USA/Argentina) and aged black garlic (USA/California) were acquired in a single, large bulk purchase.

### 2.2. Preparation of Garlic Extracts

Raw garlic was peeled and washed. Two fractions of fresh white garlic were crushed; one fraction was flash-frozen immediately in liquid nitrogen, and the other was set aside for 10 min to allow for the metabolic breakdown of alliin before submersion in liquid nitrogen. Black garlic was peeled and flash-frozen immediately. All samples were freeze-dried upon sufficient flash freezing at 262 × 10^−^^3^ mbar and −74 °C (Labconco, Kansas City, MO, USA). Freeze-drying preserves plants’ medicinal properties for storage [25]. Freeze drying of white and black garlic resulted in 31.8% and 17.7% mass reduction, respectively. The freeze-dried samples were homogenized and used to prepare extract solutions. Garlicin^®^ and Kyolic^®^ pills were crushed and sifted using a 2 mm sieve to sufficiently remove pill coatings. Garlic powder was used as sold with no other preparation.

Garlic extract solutions were prepared by mixing 500 mg garlic sources with 10 mL of either water or methanol in centrifuge tubes with a vortex mixer for 30 s. The resulting mixture was centrifuged at 4000× *g* for 10 min. The supernatant was filtered through a 1.70 um glass filter via vacuum filtration. The resulting solution gave us a stock extract of a given concentration (mg of garlic source/mL of solvent (water or alcohol). Serial dilutions of these extracts were prepared to establish a standard curve for quantification of antioxidant activity.

### 2.3. DPPH Assay

The DPPH method was based on previous studies [26]. A 0.25 mM solution of DPPH was prepared in solvent (water or methanol). In a 1.5 mL plastic cuvette, 500 µL of DPPH was mixed with 500 µL of garlic extract. After homogenizing the solution, cuvettes were placed in the dark and incubated at room temperature for 30 min. Absorbances of incubated solutions were measured at 517 nm using a UV/Vis spectrophotometer (Thermo Fisher, Waltham, MA, USA). Results were reported in terms of mg ascorbic acid equivalent (AAE) per g of dry garlic sample.

### 2.4. Hydroxyl Scavenging Assay

Hydroxyl scavenging activity of garlic extracts was determined based on previous studies [27]. Stock solutions of ethylenediaminetetraacetic acid (EDTA) (1 mM), FeCl_3_ (10 mM), ascorbic acid (1 mM), H_2_O_2_ (10 mM), and deoxyribose (10 mM) were prepared in Nanopure^TM^ water. The following solutions were added to the centrifuge tube: 0.1 mL EDTA, 0.01 mL FeCl_3_, 0.1 mL H_2_O_2_, 0.36 mL deoxyribose, 1.0 mL of sample (water used in blank), 0.33 mL of phosphate buffer (50 mM, pH 7.9) and 0.1 mL of ascorbic acid. The mixture was vortexed and then incubated for 1 h at 37 °C. From this reaction mixture, 1.0 mL was removed and mixed with 1.0 mL 10% trichloroacetic acid (TCA) to stop the reaction, 1.0 mL of 0.5% tert-butyl alcohol (TBA) was added to develop the color, and the mixture was then boiled in a water bath for 15 min. Absorbance readings were done at 532 nm after solutions returned to ambient temperature. Results were reported as percent inhibition calculated as:

$$[\frac{{Absorbance}_{532\;\mathrm{nm}\;of\;blank\;control\;}-{Absorbance}_{532\;\mathrm{nm}\;of\;sample\;}\;}{{Absorbance}_{532\mathrm{nm}\;of\;blank\;control\;}}]\times 100$$

### 2.5. Superoxide Radical Scavenging Assay

Scavenging of the superoxide (O_2_^•−^) anion radical was measured [28]. To a reaction vessel, 0.2 mg of nitroblue tetrazolium (NBT) (1 mg/mL in dimethyl sulfoxide (DMSO)), 0.6 mL of garlic sample extract and 2 mL of alkaline DMSO were added. Mixtures were vortexed for 15 s at room temperature, and absorbance was read at 560 nm. Results were reported in mg ascorbic acid equivalents per g of dry garlic sample.

### 2.6. Cell Line Selection and Maintenance

Three lung cancer cell-lines were selected to represent the most common lung cancer subtypes: H1975 adenocarcinoma cells (EGFR mutant), H520 squamous cells (TP53 mutant), and A549 adenocarcinoma cells (KRAS mutant). All cell lines were purchased from American Type Culture Collection (ATCC) (Rockville, MD, USA). Cell passage was performed roughly twice per week. The population doubling time of these cells was approximately 48 h. Cell passage was only performed on culture flasks in which cells were approximately 70% confluent. H1975 cells and H520 cells were cultured in RPMI 1640 plus 10% fetal bovine serum (Corning, 1004-CV, Corning, NY, USA) and 1% penicillin/streptomycin (Corning, 30-002-CI). A549 cells were cultured in basal medium eagle (BME) media with 10% fetal bovine serum, 0.1% penicillin/streptomycin, and glutathione. Cells were maintained at 37 °C and 5% CO_2_. All garlic extracts were diluted in cell culture media to a final concentration of 200 mg/mL of garlic extract prepared by the University of Buffalo Chemistry Department (Buffalo, NY, USA) with final concentrations: 0.15 mg/mL, 0.3125 mg/mL, 0.625 mg/mL, 1.25 mg/mL, 2.5 mg/mL, 5 mg/mL.

### 2.7. Sulforhodamine B (SRB) Assay

The antiproliferative activity of garlic forms in both solvents was determined using the SRB assay described previously [29]. The cells were seeded into 12 well plates (4 × 10^4^ cells/well for H520 and A549, 2 × 10^4^ cells/well for H1975 cells) in triplicate and allowed to attach overnight. After 24 h of incubation, the medium was replaced with a fresh growth medium in a 2-fold serial dilution series of aqueous and methanol extracts of garlic. After incubating the cells for 48 h, the cell density was measured based on cellular protein content with the SRB assay. The plate was removed from the incubator, and 0.5 mL of cold 10% TCA is added to each well and set in the cold room for 1 h. After 1 h, the plates were washed four times with tap water. The plates were left to dry then 100 µL of 0.057% SRB solution was added to each whell. The plate is left at room temperature for 30 min on a gyratory shaker and then rinsed four times 1% acetic acid to remove unbound dye. The plate was left to dry at room temperature. In order to measure the optical density, 100 µL of 10 mM Tris base solution was added to each well and placed on a gyratory shaker for 5 min to stabilize the protein-bound dye. Optical density (OD) was measured at 510 nm in a 96-well microplate reader.

Percent growth inhibition is calculated as:

$$\%\;\mathrm{of}\;\mathrm{control}\;\mathrm{cell}\;\mathrm{growth}=\frac{{mean\;OD\;}_{sample}-{mean\;OD}_{blank}}{{mean\;OD}_{control}-{mean\;OD}_{blank}}\;\times \;100$$

% growth inhibition = 100 − % of control cell growth

In confirmatory studies, the trypan blue viability method was used to determine the number of viable cells [30]. These results were compared to the findings from the SRB assay (Supplementary Figure S1).

### 2.8. Protein Extraction and Western Blot Analysis

Garlicin^®^ and fresh/raw garlic (set aside) showed the highest antiproliferative activity in alcohol and water extracts, respectively, and Kyolic^®^ water extracts showed minimal or no activity. Therefore, these three extracts were selected for the western blot analysis. H1975 cells were seeded at 2 × 10^4^ cells/well, and 24 h later, the media was replaced. Treatments were done by adding garlic extracts to culture media. Forty-eight hours later, cells were detached and harvested by collecting the culture medium. Media was aspirated, and the cell pellet was resuspended and washed in ice cold PBS. Cell pellets that were not analyzed immediately were stored at −80 °C. Protein samples were then homogenized with lysis buffer that contained protease (Millipore, Burlington, MA, USA) and phosphatase inhibitor (Calbiochem, San Diego, CA, USA). Supernatant fractions containing protein extracts were transferred into clean tubes after centrifugation for 15 min at 14,000 rpm and 4 °C. The protein concentration was determined using the BCA Protein Assay Kit (ThermoFisher Scientific, San Diego, CA, USA,). Next, 5–45 μg of total protein/sample were resolved on precast Criterion Tris/HCL protein gels (BioRad, Hercules, CA, USA). The membrane was then incubated with the appropriate dilutions of primary antibody (PARP (Cell Signaling Technology, Danvers, MA, USA, (CS); at 1:1000 dilution) in blocking buffer for a minimum of 1 h. Finally, the membranes were washed with 0.1% Tween-20 (TBST) and incubated with secondary antibody (anti-mouse 1:500) in blocking buffer. Tubulin was used as the loading control. Bands were visualized using darkroom development techniques for chemiluminescence. Immunoblots reported in the results are representative of at least three experiments that gave similar results. Densitometric of immunoreactive bands was calculated using the software Quantity One (Bio-Rad) and normalized for the loading control.

### 2.9. Data Processing and Statistical Analysis

The concentration of garlic extract plotted against the absorbance data from the standard solutions (ascorbic acid) to develop a standard curve. The strength of the linear relationships was assessed using coefficients of determination (R-squared). Absorbance readings from the garlic extracts were calculated using the standard curve to report scavenging activity in ascorbic acid equivalents (AAEs). Normalization was performed to compare activities across varying samples and varying assays. Specifically, absorbance readings were multiplied by the volume of extract used in the assay and then divided by the mass of garlic sample to yield a value of mg ascorbic acid per g of garlic sample (mg AAE per g of garlic). Antioxidant activity was expressed as ascorbic acid equivalents to make meaningful and direct comparisons with other food and plant materials examined by other authors.

For the cell culture experiments, the 50% inhibitory concentration (IC_50_) values, defined as the volume of garlic (mg/mL) that inhibits 50% of cell growth, were calculated from concentration–response curves using GraphPad Prism software. For IC_50_ calculation, the maximum was constrained to 1 and normalized to control untreated samples. Two independent experiments were performed and used for these calculations.

Data are shown as mean ± SD. Differences between garlic forms were evaluated by analysis of variance (ANOVA) tests followed by Tukey’s HSD post hoc test for multiple comparisons using GraphPad Prism software. Differences between extraction methods (alcohol compared to water) were evaluated by *t*-tests correcting for multiple comparisons using the Holm-Sidak method. Differences were considered significant at *p* < 0.05.

## 3. Results

### 3.1. DPPH Assay

**Figure 1:**
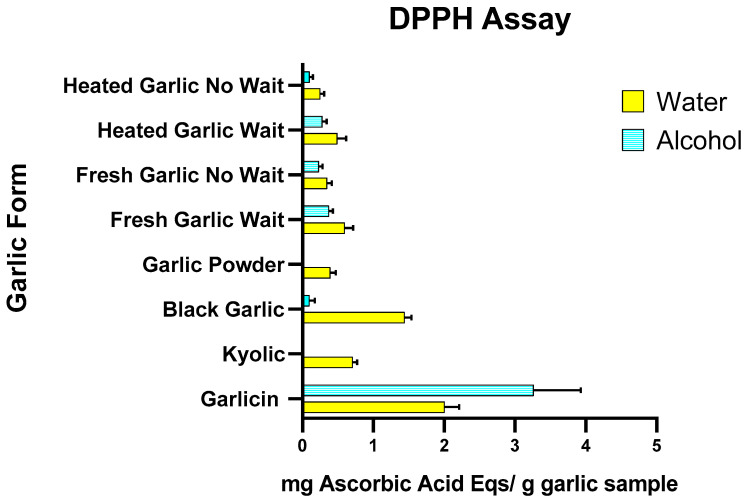
Effects of water and alcohol extract of heated/cooked garlic, heated/cooked garlic crushed and set aside for 10 min, fresh/raw garlic, fresh/raw garlic crushed and set aside for 10 min, garlic powder, black garlic, Kyolic^®^ supplement and Garlicin^®^ supplement on DPPH scavenging activity. Data are expressed as mg Ascorbic Acid equivalents per gram of garlic sample (three independent experiments for each garlic sample).

The effect of garlic extracts on radical scavenging with the DPPH assay is shown in Figure 1 and Supplementary Tables S2.1 and S2.2. Antioxidant activity ranged from 0.25 to 2.01 mg AAE per g of garlic for aqueous extracts and 0 to 3.26 mg AAE per g of garlic for methanol extracts. Of all garlic sources investigated using the DPPH assay, Garlicin^®^ showed the highest antioxidant activity in both water and methanol extracts (2.02 ± 0.22 mg AAE per g of garlic and 3.53 ± 0.26 mg AAE per g of garlic, respectively). Black garlic showed higher antioxidant activity in water compared to alcohol extract (1.44 ± 0.31 mg AAE per g of garlic and 0.08 ± 0.06 mg AAE per g of garlic, respectively). For fresh garlic, there was higher antioxidant activity in water than in the alcohol extract (0.60 ± 0.10 mg and 0.39). Heated/cooked garlic had low antioxidant activity in both alcohol and water extracts (0.25 ± 0.04 and 0.09 ± 0.01, respectively). When the fresh garlic was set aside for 10 min before being heated, it had higher antioxidant activity than if it was crushed and heated immediately (0.49 ± 0.06 and 0.28 ± 0.03, respectively). Kyolic^®^ and garlic powder seasoning showed no activity among alcohol extracts but slight activity in water extracts (0.71 ± 0.13 and 0.40 ± 0.04, respectively).

### 3.2. Superoxide Assay

**Figure 2:**
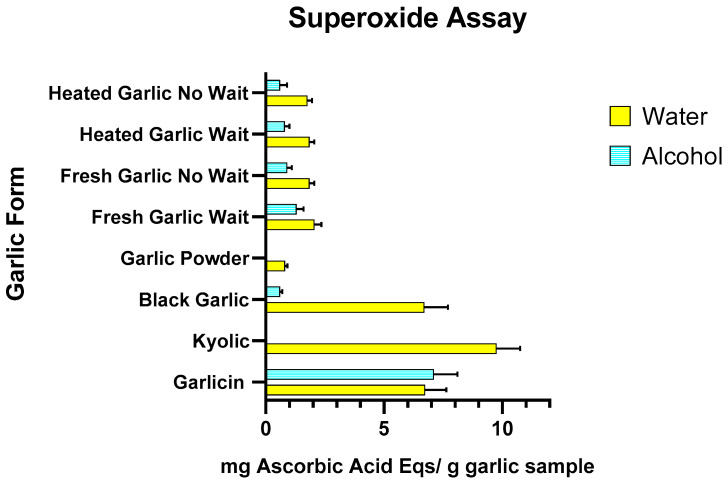
Effects of water and alcohol extract of heated/cooked garlic, heated/cooked garlic crushed and set aside for 10 min, fresh/raw garlic, fresh/raw garlic crushed and set aside for 10 min, garlic powder, black garlic, Kyolic^®^ supplement and Garlicin^®^ supplement on DPPH scavenging activity. Data are expressed as mg Ascorbic Acid equivalents per gram of garlic sample (three independent experiments for each garlic sample).

Regarding the superoxide free radical assay, the scavenging effect for the garlic forms ranged from 0.81 to 9.8 mg AAE per g of garlic for water extracts and 0 to 7.13 mg AAE per g of garlic for methanol extracts (Figure 2 and Supplementary Tables S2.3 and S2.4). The highest inhibition of both water and alcohol-based extractions were shown by Garlicin^®^ (6.73 ± 0.92 mg AAE per g of garlic and 7.13 ± 1.00 mg AAE per g of garlic, respectively). Kyolic^®^ and black garlic also showed high radical reducing capacity only in the water extracts (9.8 ± 0.95 mg AAE per g of garlic and 6.7 ± 0.99 mg AAE per g of garlic, respectively). All forms of white garlic, both cooked/heated and raw/fresh, exhibited minimal antioxidant activity. Fresh garlic set aside for 10 min exhibited higher antioxidant activity in water than in alcohol extract (2.05 ± 0.32 mg AAE per g of garlic and 1.27 ± 0.27 mg AAE per g of garlic) and higher antioxidant activity compared to fresh garlic with no waiting period (1.85 ± 0.23 mg AAE per g of garlic in water and 1.27 ± 0.27 mg AAE per g of garlic alcohol extracts). Heated garlic showed lower antioxidant activity than fresh garlic in water and alcohol extracts (1.76 ± 0.23 mg AAE per g of garlic and 0.58 ± 0.25 mg AAE per g of garlic). Garlic powder showed minimal to no activity in water and alcohol extracts (0.81 ± 0.11 mg AAE per g of garlic and 0 mg AAE per g of garlic, respectively).

### 3.3. Hydroxyl Assay

**Figure 3:**
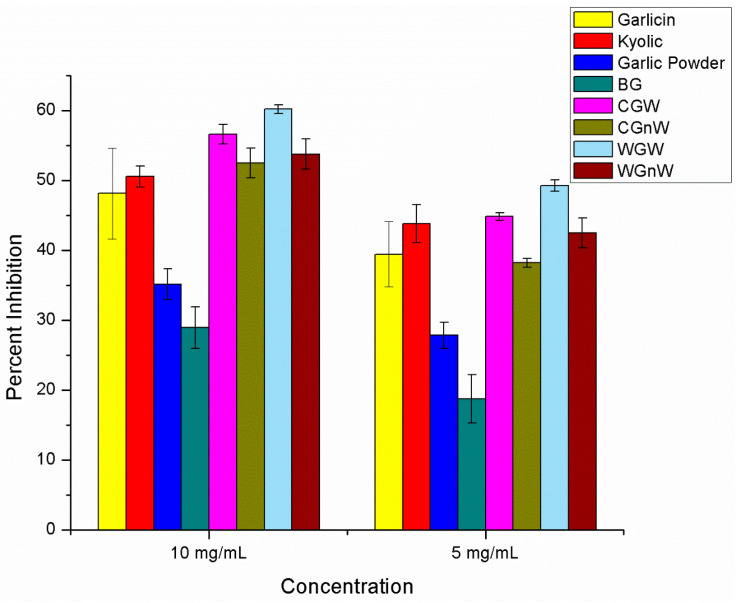
Percent inhibition of all garlic samples in hydroxyl radical scavenging assay at 10 mg/mL and 5 mg/mL water extracts (six independent experiments for each garlic sample). Abbreviations: BG = black garlic; CGW = cooked/heated garlic crushed and set aside; CGnW = cooked/heated garlic without setting it aside; WGW = white fresh/raw garlic crushed and set aside; WGnW = white fresh/raw garlic without setting it aside.

The scavenging effect of water garlic extracts on hydroxyl radicals were only examined for two concentrations, 10 mg/mL, and 5 mg/mL is shown in Figure 3 and Supplementary Tables S2.6 and S2.7. The fresh/raw garlic exhibited the greatest amount of inhibition. Fresh/raw garlic set aside for 10 min resulted in higher inhibition (60.2% ± 0.6) compared to fresh/raw garlic that was immediately treated and heated/cooked garlic (53.8% ± 2.2 and 52.5% ± 2.2, respectively). Garlicin^®^ and Kyolic^®^ pills also exhibited high inhibition (48.1% ± 6.5 and 50.6% ± 1.5, respectively). Garlic powder and black garlic exhibited the least hydroxyl radical inhibition (35.1% ± 2.2 and 28.9% 3.0, respectively).

### 3.4. Cell Proliferation Assay

**Figure 4:**
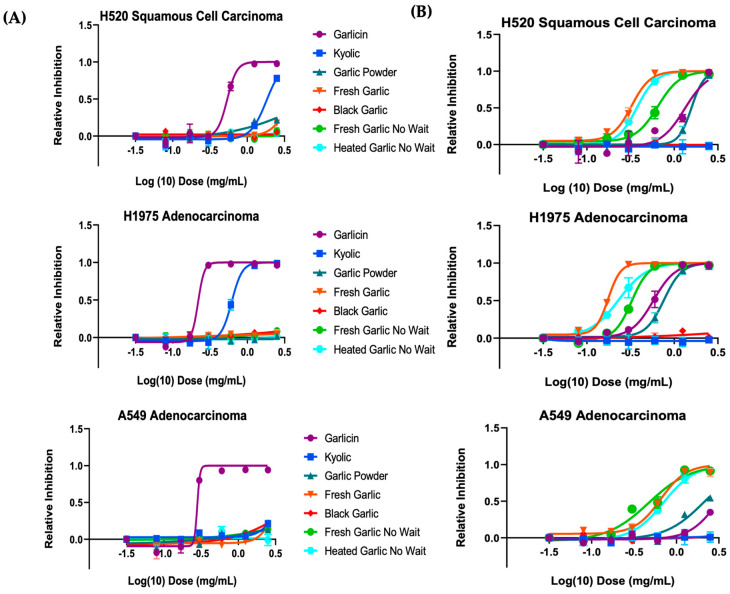
Garlicin^®^ shows potent inhibition of cells in (**A**) alcohol and (**B**) water extracts in H520, H1975, A549 cells, respectively. Dose-response curve of several garlic forms by SRB assay. Cells were treated with a range of garlic concentrations as indicated to assess the cytotoxic activity for 48 h. All values are averages of replicates expressed relative to cell viability values in untreated cells normalized to 1. Cytotoxicity curves represent two independent experiments with 3 replicates per concentration for each experiment. Vertical bars in the graphics represent ± SEM. Error bars not shown are shorter than the size of the symbol.

The SRB assay determined the effect of the different garlic extracts on lung cancer cell viability, and the results are shown in Figure 4. Dose-response inhibition is shown on a log scale. The results are expressed as the relative inhibition of garlic necessary to inhibit lung cancer cells’ proliferation by 50% relative to control cells that were not treated. Some of the garlic extracts, at final concentrations ranging from 0.15 mg/mL to 5 mg/mL, inhibited proliferation of all three lung cancer cell lines tested. Among alcohol extracts, Garlicin^®^ supplement showed potent inhibition on the growth of H1975 cells (IC_50_ = 0.22 mg/mL), H520 (IC_50_ = 0.54 mg/mL) and A549 cells (IC_50_ = 0.28 mg/mL). Among water extracts, fresh garlic that was set aside for 10 min showed potent inhibition on the growth of H1975 cells (IC_50_ = 0.16 mg/mL). Garlicin^®^ water extract also exhibited strong inhibition (IC_50_ = 0.58 mg/mL). Black garlic and garlic powder extracts induced little to no growth inhibition across all three cell lines (Figure 4). Similarly, this result was observed across H520 squamous cells and A549 adenocarcinoma cells, indicating hat the trend was mutation independent; the garlic forms have generalizable activity across lung cancer subtypes.

### 3.5. Western Blot

**Figure 5:**
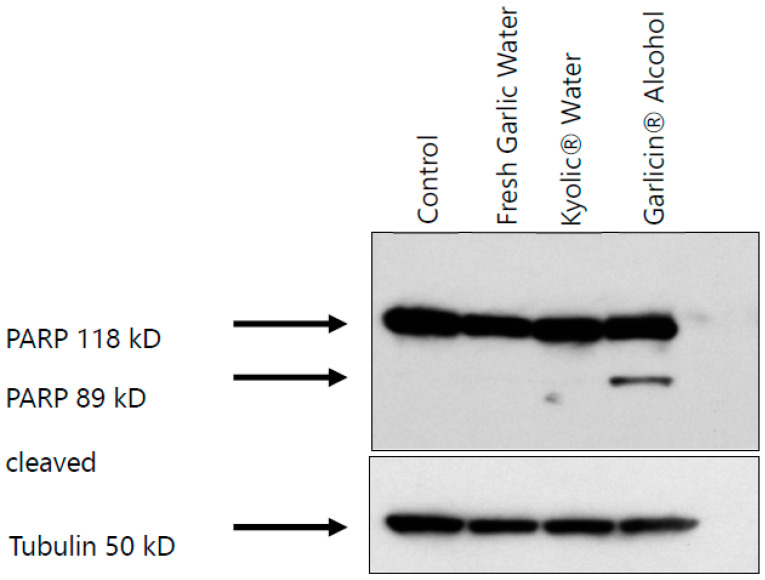
Garlicin^®^ treatment induces PARP cleavage. H1975 lung cancer cells treated with Garlicin^®^ alcohol extract, fresh garlic water extract, and Kyolic^®^ water extract for 48 h. Western blot to determine protein expression PARP cleavage. Untreated cells were used as controls (Three independent experiments for each garlic sample).

Western blots were conducted to detect the protein PARP, a nuclear polymerase involved in DNA repair in response to environmental stress. Detection of cleaved PARP-1 is a recognized general marker of apoptosis and once cleaved during apoptosis, its DNA repair function is impaired. H1975 cells were treated with fresh garlic water, Kyolic^®^ water, and Garlicin^®^ alcohol extracts (IC_50_ = 0.3125 mg/mL) for 48 h. Treatment with Garlicin^®^ extracts induced proteolytic cleavage of PARP (116 kDa), resulting in the second band at 85 kDa cleavage product (Figure 5 and Supplementary Figure S2). However, no bands were observed for treatment with Kyolic^®^ water and fresh garlic water extracts.

## 4. Discussion

Current epidemiological and clinical evidence regarding garlic and cancer prevention are inconclusive. This inconsistency is likely the result of a lack of standardization of which active ingredients of garlic are studied, the type of garlic form being assessed varies, and the differences in preparation methods of garlic (heated/cooked vs. raw). Different garlic forms do not contain the same type and quantities of the active compounds, and different garlic forms have been found to have significant antioxidant effects in animals and humans [5,7,31,32]. We compared the antioxidant and antiproliferative activity of several common garlic forms in water and methanol extracts. Across all three antioxidant assays, Garlicin^®^ showed high antioxidant activity in both water and alcohol-based extractions. Garlicin^®^ also showed potent inhibition across lung cancer cells independent of the cell line in alcohol-based extractions, while fresh garlic showed potent inhibition in water-based extractions. Furthermore, methanol extract of Garlicin^®^ led to induction of apoptosis in H1975 lung cancer cells. Garlic powder and black garlic displayed minimal antioxidant and antiproliferative activity.

Spectrophotometric techniques are commonly used for measuring the antioxidant activity of foods. In general, studies use more than one spectrophotometric method to determine the synthetic/natural antioxidant profile of foods. Previous studies measuring this antioxidant activity have utilized assays, including the DPPH assay, the 2,2′-azinobis-(3-ethylbenzothiazoline-6-sulfonate (ABTS) assay, ferric reducing antioxidant power (FRAP) assay, copper-chelating assay, iron chelating assay, superoxide scavenging assay, and hydroxyl radical scavenging assay [33,34,35]. Antioxidants act by several mechanisms, and no single assay can capture all the different modes of action of antioxidants [36]. Of all garlic sources that we investigated across the antioxidant assays, Garlicin^®^ consistently showed high levels of antioxidant activity. Prasad et al. previously observed that Garlicin^®^ also reported high antioxidant activity using the DPPH and superoxide assay [37].

Fresh/raw garlic also exhibited potent antioxidant activity, especially in the hydroxyl scavenging assay. It contains water-soluble compounds as well as a variety of oil-soluble sulfur compounds. Fresh raw garlic has low allicin content when it is ingested, as the compound rapidly breaks down into other sulfur compounds. Fresh/raw white garlic with a 10-min wait period, as expected, had higher antioxidant activity than fresh/raw garlic that was crushed and immediately submerged into liquid nitrogen. This may be due to the oxygen-induced conversion of allicin into a wider array of compounds, including diallyl sulfide (DAS), diallyl disulfide (DADS), and diallyl trisulfide, with increased antioxidant and antiproliferative activity compared to fresh garlic (not set aside for 10 min). With heating of both water and methanol extracts, there was depletion of antioxidant and antiproliferative activity of the fresh garlic. Cooking or even heating garlic may reduce some of the bioactive compounds present in garlic [17].

There was low antioxidant activity for garlic powder. The preparation of garlic powder typically involves chopping of garlic, followed by heating to approximately 150 °C. Once all water is removed, the dehydrated garlic is minced to the desired particle size. The relative instability of the OSCs in garlic, both at high temperatures as well as with exposure to air, may explain the lower antioxidant activity [38]. Black garlic is a thermally treated garlic which allows the natural sweetness of its sugars to dominate its flavor profile. Chemically, the result is a large variety of organosulfur compounds which are transformation products of allicin. These products are more polar than allicin, explaining the high antioxidant activity of the water-based extraction in comparison to the more nonpolar solvents [39].

Garlicin^®^ methanol extract also exhibited antiproliferative effects on three common lung cancer cell lines. In previous studies, isolated allicin has been shown to inhibit the growth of cancer cells more than some of the derived organosulfur compounds including SAMC, DAS, DADS, DATS, SAC, and ajoene [40]. Previous studies have focused on using isolated fat-soluble compounds such as allylsulfides or water-soluble compounds such as SAC to study the anticarcinogenic effect of garlic [41,42,43]. However, extracts contain several bioactive molecules and are more representative of the normal consumption of these products.

In our study, treatment of H1975 adenocarcinoma cells with Garlicin^®^ induced the cleavage of PARP, a known marker for apoptosis, since there was a second band at 89 kD. Previously, metabolites of allicin such as diallyl disulfide and diallyl trisulfide have been shown to induce apoptosis in leukemia HL-60 cells [44]. Other metabolites, such as ajoene and S-allylmercaptocysteine, have also been shown to induce apoptosis in human promyeloleukemic and erythroleukemia cell lines, respectively [45,46]. The molecular mechanisms resulting in apoptosis after treating cells with Garlicin^®^ are only partly understood, and further studies of this process are important.

We found that processing methods of garlic result in quite different antioxidant and antiproliferative effects. There is currently no credible evidence to support an effect of garlic intake or supplement use on lung cancer risk. The chemistry of garlic is complex, with a variety of volatile and non-volatile OSCs, aromatics, nitriles, and esters that may play important roles in the beneficial effects of garlic [47]. In prospective studies, no association of garlic intake and garlic supplement use was observed with lung cancer risk [21,48]. However, raw garlic consumption at least two times/week was associated with a 50% reduced risk of lung cancer in case-control studies [20,49,50]. Additional studies on garlic and cancer are needed with information on the preparation method used (whether the garlic is raw or cooked, whole, or extracted).

Our study has some limitations. First, in vitro study of garlic extracts does not necessarily predict the in vivo effects in that it does not include the processing of food within the gastrointestinal tract. Further studies in animals and/or humans would be needed to investigate garlic extracts’ effects in vivo. Secondly, we used microwaved garlic to simulate the effect of heating garlic, which may overestimate the antioxidant and antiproliferative activity of garlic compared to oven-heated techniques. Boiling, deep frying, and pan-frying garlic in a hot cooking medium can reduce antioxidant activity [51,52]. Finally, we were not able to isolate the compounds for further examination; additional studies may be needed to examine the chemical characterization of compounds. Our study’s strengths include the standardized comparison of several commonly used garlic forms rather than just examining one individual garlic compound. Secondly, we studied extracts of garlic rather than individual compounds to represent actual consumption within the gastrointestinal tract. Third, we measured two important biologically relevant radicals, hydroxyl radical and superoxide anion, that are produced within the human body. Previous studies have been limited to artificially generated radicals, such as reducing ferric ion using the ferric reducing-antioxidant power (FRAP) assay. Finally, the in vitro study was conducted across three of the most common lung cancer subtypes indicating that garlic forms have generalizable activity across lung cancer subtypes.

## 5. Conclusions

In conclusion, we found that Garlicin^®^, a commercially available garlic supplement, is a promising source of antioxidant and antiproliferative activity. We also observed that fresh/raw garlic water extracts had high antiproliferative activity against all three cell lines. To our knowledge, ours is the first study to compare several common forms and preparation methods of garlic across several antioxidant and cancer cell lines in aqueous and methanol extracts. We also found that heating garlic or garlic powder may reduce the antioxidant and anticancer activity of garlic. These findings provide evidence that different garlic preparations have different pharmacologic properties. Future epidemiological studies of garlic associations with disease risk should include the variability of garlic consumption including consideration of factors such as cooking or processing methods, which may affect the biological properties of compounds in garlic.

## Data Availability

Not applicable.

## Supplementary Materials

The following supporting information can be downloaded at: https://www.mdpi.com/article/10.3390/nu15194099/s1, Supplementary Table S1.1: Relative inhibition of garlic forms in H520, H1975, A549 lung cell lines (mean ± SE); Supplementary Table S1.2: Results of ANOVA comparing different garlic forms in alcohol in H520 lung cancer cells; Supplementary Table S1.3: Results of ANOVA comparing different garlic forms in water in H520 lung cancer cells; Supplementary Table S1.4: Results of ANOVA comparing different garlic forms in alcohol in H1975 lung cancer cells; Supplementary Table S1.5: Results of ANOVA comparing different garlic forms in water in H1975 lung cancer cells; Supplementary Table S1.6: Results of ANOVA comparing different garlic forms in alcohol in A549 lung cancer cells; Supplementary Table S1.7: Results of ANOVA comparing different garlic forms in water in A549 lung cancer cells; Supplementary Table S2.1: Antioxidant activity of several garlic forms in water and alcohol extracts (mean ± SD); Supplementary Table S2.2: Results of ANOVAa comparing DPPH activity for different garlic forms in water; Supplementary Table S2.3: Results of ANOVAa comparing DPPH activity for different garlic forms in alcohol; Supplementary Table S2.4: Results of ANOVAa comparing superoxide activity for different garlic forms in water; Supplementary Table S2.5: Results of ANOVAa comparing superoxide activity for different garlic forms in alcohol; Supplementary Table S2.6: Results of ANOVAa comparing hydroxyl activity for different garlic forms at 5 mg/mL; Supplementary Table S2.7: Results of ANOVAa comparing hydroxyl activity for different garlic forms at 10 mg/mL; Supplementary Figure S1: Effect of garlic extracts on cell proliferation determined by the trypan blue method. Supplementary Figure S2: Garlicin™ treatment induces PARP cleavage.
