# Supplementary material for: Antioxidant and Antiproliferative Activities of Several Garlic Forms
Source: Nutrients. 2023 Sep 22;15(19):4099. doi: 10.3390/nu15194099 (PMC10574068; doi:10.3390/nu15194099)
Supplement: Supplementary file 1 [file nutrients-15-04099-s001.zip › nutrients-2543788-supplementary.pdf]

Supplemental File:

Antioxidant and Antiproliferative Activities of Several Garlic Forms

Zeinab Farhat [1], Tyler Scheving [2], Diana S. Aga [2], Pamela A. Hershberger [3], Jo L. Freudenheim [1], Rachael Hageman Blair [4], Manoj J. Mammen [5] and Lina Mu [1,*]

[1] Department of Epidemiology and Environmental Health, State University of New York at Buffalo, Buffalo, NY 14260, USA; zfarhat@buffalo.edu (Z.F.); jfreuden@buffalo.edu (J.L.F.)
[2] Department of Chemistry, College of Arts and Sciences, State University of New York at Buffalo, Buffalo, NY 14260, USA; tjschevi@buffalo.edu (T.S.); dianaaga@buffalo.edu (D.S.A.)
[3] Department of Pharmacology and Therapeutics, Roswell Park Comprehensive Cancer Center, Buffalo, NY 14263, USA; pamela.hershberger@roswellpark.org
[4] Department of Biostatistics, State University of New York at Buffalo, Buffalo, NY 14260, USA; hageman@buffalo.edu
[5] Department of Medicine, State University of New York at Buffalo, Buffalo, NY 14260, USA; mammen@buffalo.edu
***** Correspondence: linamu@buffalo.edu; Tel.: +1-(716)-829-5381

Supplementary Table S1.1: Relative inhibition of garlic forms in H520, H1975, A549 lung cell lines (mean ± SE).

	Mean ± SD*,±					
	H520		H1975		A549	
Garlic Form	Water	Alcohol	Water	Alcohol	Water	Alcohol
Garlicin	0.19 ± 0.15[a]	0.37 ± 0.18[a]	0.37 ± 0.17[a]	0.54 ± 0.20[a]	0.05 ± 0.05[a]	0.48 ± 0.20[a]
Kyolic	0.00 ± 0.01[a]	0.10 ± 0.12[a]	0.00 ± 0.0[a]	0.32 ± 0.18[a]	0.00 ± 0.01[a]	0.06 ± 0.03[a]
Fresh Garlic (wait)	0.51 ± 0.17[a]	0.03 ± 0.03[b]	0.64 ± 0.17[a]	0.02 ± 0.01[b]	0.37 ± 0.15[a]	0.00 ± 0.04[b]
Fresh Garlic (no wait)	0.37 ± 0.16[a]	0.01 ± 0.01[b]	0.47 ± 0.18[a]	0.02 ± 0.01[b]	0.39 ± 0.16[a]	0.04 ±0.02[a]
Garlic Powder	0.16 ± 0.13[a]	0.05 ± 0.04[a]	0.32 ± 0.16[a]	0.00 ± 0.01[a]	0.12 ± 0.09[a]	0.01 ± 0.03[a]
Black Garlic	0.00 ± 0.00[a]	0.02 ± 0.01[a]	0.02 ± 0.01[a]	0.02 ± 0.01[a]	0.00 ± 0.00[a]	0.01 ± 0.04[a]
Heated Garlic	0.45 ± 0.18[a]	0.00 ± 0.01[b]	0.57 ± 0.16 [a]	0.00 ± 0.01[b]	0.30 ± 0.15[a]	0.01 ± 0.02[a]

*Different letters within a row (w–v) are significantly different (P < 0.05).

±T-test statistical significance determined using False Discovery Rate (FDR) approach (Q<0.05).

Supplementary Table S1.2: Results of ANOVA comparing different garlic forms in alcohol in H520 lung cancer cells.

	Garlicin	Kyolic	FG (wait)	FG (no wait)	Garlic powder	Black garlic	HG (no wait)
Garlicin		ns	ns	ns	ns	ns	ns
Kyolic			ns	ns	ns	ns	ns
FG (wait)				ns		ns	ns
FG (no wait)							ns
Garlic powder			ns	ns		ns	ns
Black garlic				ns			ns

[a] “ns” P>0.05, “*” Indicates P≤0.05, “**” P≤0.01, “***” P≤0.0001”, “****” P≤0.0001

Abbreviations: FG (Fresh Garlic); HG (Heated Garlic); NS (non-significant)

Supplementary Table S1.3: Results of ANOVA comparing different garlic forms in water in H520 lung cancer cells.

	Garlicin	Kyolic	FG (wait)	FG (no wait)	Garlic powder	Black garlic	HG (no wait)
Garlicin		ns	ns	ns	ns	ns	ns
Kyolic			ns	ns	ns	ns	ns
FG (wait)				ns		ns	ns
FG (no wait)							ns
Garlic powder			ns	ns		ns	ns
Black garlic				ns			ns

[a] “ns” $P>0.05$, “*” Indicates $P\leq0.05$, “**” $P\leq0.01$, “***” $P\leq0.0001$”, “****” $P\leq0.0001$

Abbreviations: FG (Fresh Garlic); HG (Heated Garlic); NS (non-significant)

Supplementary Table S1.4: Results of ANOVA comparing different garlic forms in alcohol in H1975 lung cancer cells.

	Garlicin	Kyolic	FG (wait)	FG (no wait)	Garlic powder	Black garlic	HG (no wait)
Garlicin		ns	*	*	**	*	**
Kyolic			ns	ns	ns	ns	ns
FG (wait)				ns		ns	ns
FG (no wait)							ns
Garlic powder			ns	ns		ns	ns
Black garlic				ns			ns

[a] “ns” P>0.05, “*” Indicates P≤0.05, “**” P≤0.01, “***” P≤0.0001”, “****” P≤0.0001
Abbreviations: FG (Fresh Garlic); HG (Heated Garlic); NS (non-significant)

Supplementary Table S1.5: Results of ANOVA comparing different garlic forms in water in H1975 lung cancer cells.

	Garlicin	Kyolic	FG (wait)	FG (no wait)	Garlic powder	Black garlic	HG (no wait)
Garlicin		ns	ns	ns	ns	ns	ns
Kyolic			*	ns	ns	ns	ns
FG (wait)				ns		ns	ns
FG (no wait)							ns
Garlic powder			ns	ns		ns	ns
Black garlic				ns			ns

[a] “ns” $P>0.05$, “*” Indicates $P\leq0.05$, “**” $P\leq0.01$, “***” $P\leq0.0001$”, “****” $P\leq0.0001$

Abbreviations: FG (Fresh Garlic); HG (Heated Garlic); NS (non-significant)

Supplementary Table S1.6: Results of ANOVA comparing different garlic forms in alcohol in A549 lung cancer cells.

	Garlicin	Kyolic	FG (wait)	FG (no wait)	Garlic powder	Black garlic	HG (no wait)
Garlicin		*	**	**	**	**	**
Kyolic			ns	ns	ns	ns	ns
FG (wait)				ns		ns	ns
FG (no wait)							ns
Garlic powder			ns	ns		ns	ns
Black garlic				ns			ns

[a] “ns” $P>0.05$, “*” Indicates $P\leq0.05$, “**” $P\leq0.01$, “***” $P\leq0.0001$”, “****” $P\leq0.0001$

Abbreviations: FG (Fresh Garlic); HG (Heated Garlic); NS (non-significant)

Supplementary Table S1.7: Results of ANOVA comparing different garlic forms in water in A549 lung cancer cells.

	Garlicin	Kyolic	FG (wait)	FG (no wait)	Garlic powder	Black garlic	HG (no wait)
Garlicin		ns	ns	ns	ns	ns	ns
Kyolic			ns	ns	ns	ns	ns
FG (wait)				ns		ns	ns
FG (no wait)							ns
Garlic powder			ns	ns		ns	ns
Black garlic				ns			ns

[a] “ns” P>0.05, “*” Indicates P≤0.05, “**” P≤0.01, “***” P≤0.0001”, “****” P≤0.0001
Abbreviations: FG (Fresh Garlic); HG (Heated Garlic); NS (non-significant)

Supplementary Table S2.1: Antioxidant activity of several garlic forms in water and alcohol extracts (mean ± SD).

	Antioxidant inhibition[*,±]					
	DPPH[↑]		Superoxide[↑]		Hydroxyl[ϒ]	
Garlic Form	Water	Alcohol	Water	Alcohol	10 mg/mL	5 mg/mL
Garlicin	2.02 ± 0.22[a]	3.53 ± 0.26[a]	6.73 ± 0.92[a]	7.13 ± 1.00[a]	48.1% ± 6.5[a]	39.5% ± 4.6[b]
Kyolic	0.71 ± 0.13[a]	0[b]	9.8 ± 0.95[a]	0[b]	50.6% ± 1.5[a]	43.8% ± 2.7[b]
Fresh Garlic (wait)	0.60 ± 0.10[a]	0.39 ± 0.06[a]	2.05 ± 0.32[a]	1.27 ± 0.27[b]	60.2% ± 0.6[a]	49.3% ± 0.8[b]
Fresh Garlic (no wait)	0.36 ± 0.04[a]	0.21 ± 0.05[a]	1.85 ± 0.23[a]	0.94 ± 0.18[b]	53.8% ± 2.2[a]	42.5% ± 2.2[b]
Garlic Powder	0.40 ± 0.04[a]	0[b]	0.81 ± 0.11[a]	0[b]	35.1% ± 2.2[a]	27.8% ± 1.9[b]
Black Garlic	1.44 ± 0.31[a]	0.08 ± 0.06[b]	6.7 ± 0.99[a]	0.57 ± 0.1[b]	28.9% ± 3.0[a]	18.8% ± 3.4[b]
Heated Garlic	0.25 ± 0.04[a]	0.09 ± 0.01[b]	1.76 ± 0.23[a]	0.58 ± 0.25[b]	52.5% ± 2.2[a]	38.2% ± 0.6[b]
Heated Garlic (wait)	0.49 ± 0.06[a]	0.28 ± 0.03[a]	1.85 ± 0.15[a]	0.77 ± 0.17[b]	56.6% ± 1.4[a]	44.8% ± 0.5[b]

*Different letters within a row (a-b) are significantly different ($P < 0.05$) between alcohol and water extracts.
[ϒ]Percent inhibition values for hydroxyl assays and mean mg Ascorbic equivalent per g of sample.
[±]T-test statistical significance determined using False Discovery Rate (FDR) approach ($Q<0.05$).

Supplementary Table S2.2: Results of ANOVA[a] comparing DPPH activity for different garlic forms in water.

	Garlicin	Kyolic	FG (wait)	FG (no wait)	Garlic powder	Black garlic	HG	HG (wait)
Garlicin		****	****	****	****	***	****	****
Kyolic			****	****	****	****	****	****
FG (wait)				ns			*	ns
FG (no wait)							ns	ns
Garlic powder			ns	ns			ns	ns
Black garlic			****	****	****		****	****
HG (no wait)								ns

[a] "ns" P>0.05, "*" Indicates P≤0.05, "**" P≤0.01, "***" P≤0.0001", "****" P≤0.0001
Abbreviations: FG (Fresh Garlic); HG (Heated Garlic); NS (non-significant)

Supplementary Table S2.3: Results of ANOVA[a] comparing DPPH activity for different garlic forms in alcohol.

	Garlicin	Kyolic	FG (wait)	FG (no wait)	Garlic powder	Black garlic	HG	HG (wait)
Garlicin		****	****	****	****	****	****	****
Kyolic			ns	ns	ns	ns	ns	ns
FG (wait)				ns			ns	ns
FG (no wait)							ns	ns
Garlic powder			ns	ns			ns	ns
Black garlic			ns	ns	ns		ns	ns
HG (no wait)								ns

[a] "ns" P>0.05, "*" Indicates P≤0.05, "**" P≤0.01, "***" P≤0.0001", "****" P≤0.0001
Abbreviations: FG (Fresh Garlic); HG (Heated Garlic); NS (non-significant)

Supplementary Table S2.4: Results of ANOVA[a] comparing superoxide activity for different garlic forms in water.

	Garlicin	Kyolic	FG (wait)	FG (no wait)	Garlic powder	Black garlic	HG	HG (wait)
Garlicin		***	****	****	****	ns	****	****
Kyolic			****	****	****	***	****	****
FG (wait)				ns			ns	ns
FG (no wait)							ns	ns
Garlic powder			ns	ns			ns	ns
Black garlic			****	****	****		****	****
HG (no wait)								ns

[a] “ns” P>0.05, “*” Indicates P≤0.05, “**” P≤0.01, “***” P≤0.0001”, “****” P≤0.0001
Abbreviations: FG (Fresh Garlic); HG (Heated Garlic); NS (non-significant)

Supplementary Table S2.5: Results of ANOVA[a] comparing superoxide activity for different garlic forms in alcohol.

	Garlicin	Kyolic	FG (wait)	FG (no wait)	Garlic powder	Black garlic	HG	HG (wait)
Garlicin		****	****	****	****	****	****	****
Kyolic			*	ns	ns	ns	ns	ns
FG (wait)				ns			ns	ns
FG (no wait)							ns	ns
Garlic powder			*	ns			ns	ns
Black garlic			ns	ns	ns		ns	ns
HG (no wait)								ns

[a] “ns” P>0.05, “*” Indicates P≤0.05, “**” P≤0.01, “***” P≤0.0001”, “****” P≤0.0001
Abbreviations: FG (Fresh Garlic); HG (Heated Garlic); NS (non-significant)

Supplementary Table S2.6: Results of ANOVA[a] comparing hydroxyl activity for different garlic forms at 5 mg/ml.

	Garlicin	Kyolic	FG (wait)	FG (no wait)	Garlic powder	Black garlic	HG	HG (wait)
Garlicin		ns	****	ns	****	****	ns	*
Kyolic			**	ns	****	****	**	ns
FG (wait)				***			****	*
FG (no wait)							ns	ns
Garlic powder			****	****			****	****
Black garlic			****	****	****		****	****
HG (no wait)								***

[a] “ns” P>0.05, “*” Indicates P≤0.05, “**” P≤0.01, “***” P≤0.0001”, “****” P≤0.0001
Abbreviations: FG (Fresh Garlic); HG (Heated Garlic); NS (non-significant)

Supplementary Table S2.7: Results of ANOVA[a] comparing hydroxyl activity for different garlic forms at 10 mg/mL.

	Garlicin	Kyolic	FG (wait)	FG (no wait)	Garlic powder	Black garlic	HG	HG (wait)
Garlicin		ns	****	**	****	****	ns	***
Kyolic			****	ns	****	****	ns	*
FG (wait)				*			***	ns
FG (no wait)							ns	ns
Garlic powder			****	****			****	****
Black garlic			****	****	*		****	****
HG (no wait)								ns

[a] “ns” P>0.05, “*” Indicates P≤0.05, “**” P≤0.01, “***” P≤0.0001”, “****” P≤0.0001
Abbreviations: FG (Fresh Garlic); HG (Heated Garlic); NS (non-significant)

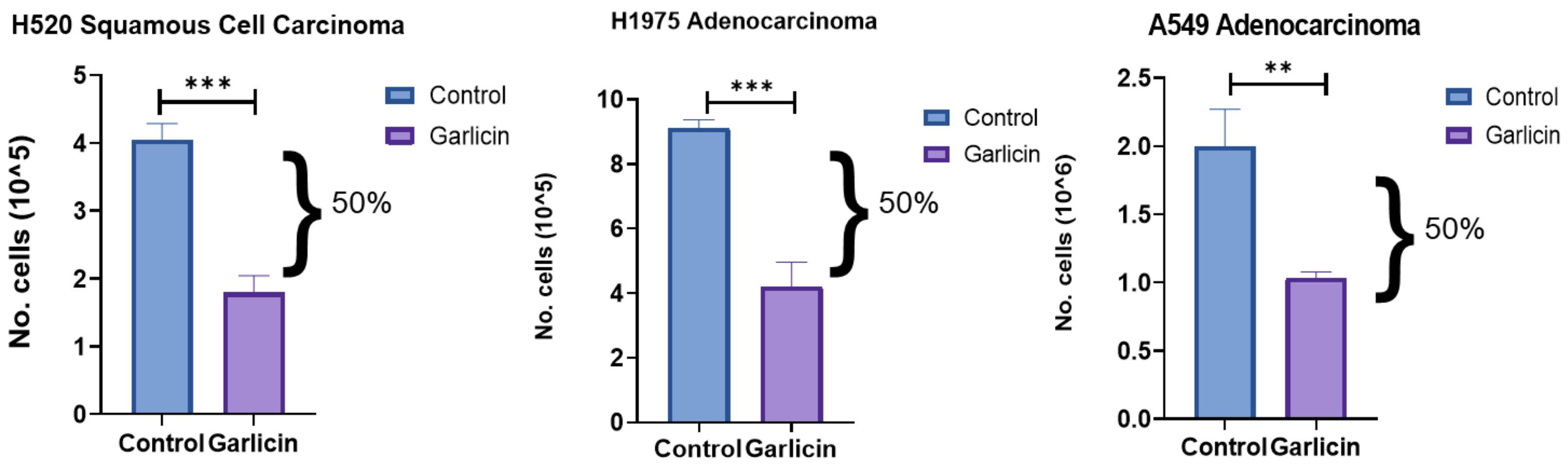

Supplementary Figure S1: Effect of garlic extracts on cell proliferation determined by the trypan blue method. Garlicin alcohol extract treated cells with IC_{50} resulted in 50% decrease in cell number. The cell viability was determined by the trypan blue exclusion method after Garlicin treatment for 48 hours. The graph represents the data from three sample replicates and the error bars indicate the standard error of the mean. "ns" P>0.05, "*" Indicates P≤0.05, "**" P≤0.01, "***" P≤0.0001", "****" P≤0.0001

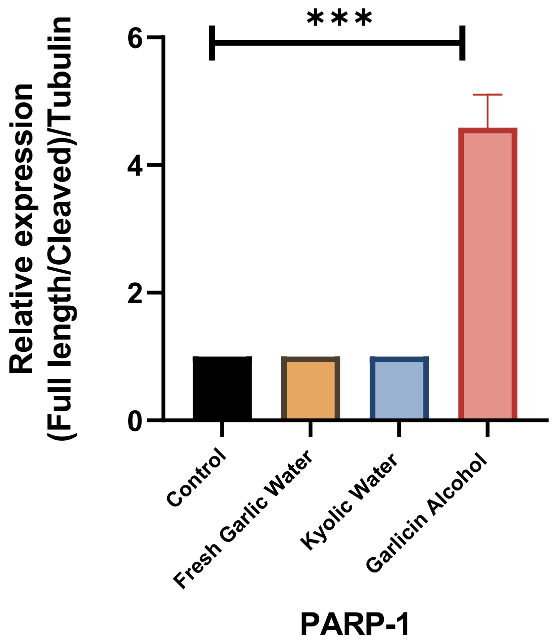

Supplementary Figure S2: Garlicin™ treatment induces PARP cleavage. H1975 lung cancer cells treated with Garlicin™ alcohol extract, fresh garlic water extract, and kyolic water extract for 48 hours. Data represent the mean ± SD of three independent experiments (bars, SD). *"ns" P>0.05, "*" Indicates P≤0.05, "**" P≤0.01, "***" P≤0.0001", "****" P≤0.0001.
